# The impact of financial toxicity on adolescent/young adult cancer survivors (AYACS): A mixed methods study

**DOI:** 10.1007/s00520-026-11093-z

**Published:** 2026-09-02

**Authors:** Pooja Rao, Kristin Bingen, Katie A. Devine, Joel E. Segel, Emily Wasserman, Courtney Rumbaugh, Tonya S. King, Heather J. Costigan, Allison M. Scott, Smita Dandekar, Kevin Rakszawski, Natthapol Songdej, George F. Blackall, Eugene J. Lengerich, Lauren J. Van Scoy

**Affiliations:** 1 Division of Pediatric Hematology/Oncology, Department of Pediatrics, Penn State Health Children’s Hospital, Hershey, PA USA; 2 Division of Pediatric Psychology and Developmental Medicine, Department of Pediatrics, Medical College of Wisconsin, Milwaukee, WI USA; 3 Rutgers Cancer Institute, New Brunswick, NJ USA; 4 Department of Health Policy and Administration, The Pennsylvania State University, University Park, PA USA; 5 Penn State College of Medicine, Hershey, PA USA; 6 Kolschowsky Research & Education Institute, Sarasota Memorial Health Care System, Sarasota, FL USA; 7 Department of Communication, University of Kentucky, Lexington, KY USA; 8 Penn State Cancer Institute, Hershey, PA USA

**Keywords:** Adolescent, Young Adult, Cancer Survivor, Financial Toxicity

## Abstract

**Purpose:**

Financial toxicity (the financial burden that accompanies a cancer diagnosis) uniquely affects adolescent/young adult cancer survivors (AYACS). The objective of this study was to use a mixed-method approach to understand AYACS’ perspectives on financial consequences of cancer treatment, to complement quantitative measures of financial toxicity.

**Methods:**

In this convergent mixed methods study, data from AYACS participants (*n* = 35) were collected. Financial toxicity was measured quantitatively using the Comprehensive Score for Financial Toxicity (COST) and a sociodemographic survey. Qualitative measures included semistructured interviews.

**Results:**

The mean COST score was 30.4 (SD 7.26), ranging from 16 to 43. Mixed methods analysis revealed: 1) financial situations related to cancer treatment led to varying levels of support and conversation within families; 2) financial strain was significant for participants of all ages and impacted future planning; 3) participants experienced gratitude for philanthropic funding, while experiences with insurance were mixed; and 4) due to financial strain, some participants experienced stressful emotions, while others did not. No statistically significant differences were found in COST scores across demographic variables.

**Conclusions:**

AYACS experienced financial challenges. While quantitative data did not reveal statistically significant differences in financial toxicity by sex, diagnosis age, or treatment phase, qualitative data uncovered important differences in experiences of younger versus older AYACS, particularly social support’s role in protecting against financial toxicity. This study revealed the importance of improving services to ensure AYACS (especially older AYACS) have resources to decrease financial toxicity and designing sensitive quantitative instruments to assess financial toxicity among AYACS with additional focus on assessing social aspects of financial support.

**Supplementary Information:**

The online version contains supplementary material available at https://doi.org/10.1007/s00520-026-11093-z.

## Introduction

Adolescent/young adult cancer survivors (AYACS) are people diagnosed with cancer between 15 and 39 years of age. While treatment improvements have resulted in survival rates of 80–90% for AYACS [1], a source of adversity is financial toxicity, the financial burden that accompanies a cancer diagnosis [2, 3]. While cancer survivors including AYACS experience financial toxicity (e.g., devoting significant income to medical costs and costs of daily living [4–8]), young adulthood is a starting time for many individuals’ lifetime earnings, with unique and long-lasting disruptive effects for AYACS [9–12]. Financial toxicity affects AYACS’ professional choices related to “job lock”—staying at a job to keep work-related health insurance [13]. Financial toxicity also has detrimental impacts on AYACS’ psychological health [14], with these costs further increasing financial toxicity [15].

The Comprehensive Score for Financial Toxicity (COST) is a validated measure of patient-reported outcomes of financial toxicity for cancer survivors [16, 17]. While used among AYACS [18, 19], COST may not capture AYACS’ unique financial challenges like financial independence, social, professional, and psychological consequences of financial toxicity [12, 20]. While there is interest to develop an AYA-specific financial toxicity measure, none are currently available [11]. The objective of this study was to use a mixed-method approach to understand AYACS’ perspectives on financial consequences of cancer treatment, to further complement quantitative financial toxicity measures using COST.

## Methods

### Study population

This mixed method analysis was part of a larger study assessing AYACS’ psychosocial challenges. Participants were AYACS 15–25 years old at time of cancer diagnosis and within six years of cancer diagnosis. This subset was chosen to focus on those undergoing similar challenges (i.e., higher education completion, shaping career goals, and planning financial independence) [21]. Participants were receiving or had received oncologic care at Penn State Health, an academic tertiary care center. Participants were fluent in written and spoken English and had computer or smartphone access. Participants who were non-English speaking, with relapsed cancer, or with cognitive or physical inability to participate (determined by study team or participant’s oncology team) were excluded.

### Recruitment

Data collection occurred Winter 2021–Summer 2022, with remote recruitment conducted until early 2022, when COVID-19 study precautions lifted and in-person recruitment renewed. Participants were identified through review of the institutional cancer registry, divisional cancer database, electronic medical record (EMR), and clinic templates. Enrollment included representation of participants of two age groups at cancer diagnosis (i.e., 15–21 years old or 22–25 years old) and treatment phases (i.e., receiving or completed cancer treatment). The age threshold of 21 years old was chosen due to our institution’s philanthropic funding for patients diagnosed with cancer < 22 years old, which cover costs of supportive care services, financial vouchers, medication, and test costs not covered by insurance.

We expected data saturation at 20 interviews based on our pilot work [22] but aimed to recruit 35–40 participants to ensure adequate representation by age and cancer treatment phase.

### Qualitative data collection

Demographic data was collected through a participant questionnaire and EMR review. Quantitative data about financial toxicity was collected using COST [16, 17]. Respondents rate items on a 5-point Likert scale for 11 items, yielding a 0–44 composite score. Lower scores suggest worse financial toxicity. While COST was validated in patients ≥ 18 years old with cancer [16, 17], COST has been used with AYACS [18, 23]. Thresholds indicate clinically meaningful differences in financial toxicity severity (0–13 = severe, 14–25 = moderate, and > 25 = none to little) [24].

### Qualitative data collection

Two qualitative interviewers utilized a semi-structured interview guide (Appendix 1) to conduct individual videoconference interviews. The interview guide was created based on literature review and informal AYACS conversations about financial aspects of cancer treatment. Interviews lasted 30–45 min, were audio-recorded, and transcribed verbatim by a professional transcription service.

### Quantitative data analysis

Descriptive statistics for demographic characteristics were summarized using mean, standard deviation, median, and ranges for continuous measures, and frequency counts with percentages for categorical measures. Proportions of participants with none to minimal financial toxicity (COST score over 25), moderate financial toxicity (14–25), and severe financial toxicity (0–13) were calculated [23]. Two-sample independent *t*-tests assuming equal group variances were used to compare differences in continuous COST scores by sex (female vs. male), cancer diagnosis age (15–21 years vs. 22–25 years), and treatment phase (completed vs. receiving treatment). Fisher’s exact test was used to compare differences in proportions of COST financial toxicity categories by sex, cancer diagnosis age, and treatment phase. A significance level of 0.05 was used for all statistical tests. All statistical programming and analyses were performed using SAS software Version 9.4 (SAS Institute Inc., Cary, NC, USA).

### Qualitative data analysis

Thematic analysis was used to analyze interview data using a phenomenological approach to explore AYACS’ experiences related to financial consequences after a personal cancer diagnosis. A phenomenological approach seeks to understand a phenomenon by exploring it from the perspective of those who experienced it [25] and avoids preconceived categories. It is appropriate to use when literature on a topic is limited [26]. MAXQDA software [27] was used to organize and manage data. Experienced qualitative analysts reviewed approximately 10–20% of the total dataset to inductively create preliminary categories, codes, and subcodes describing the data. A preliminary codebook related to “Finances” and its related codes was constructed and used to code five transcripts after which discrepant codes were adjudicated by group discussion to create a final codebook with codes, definitions, and exemplars. Two coders then independently coded the entire dataset using the constant comparison method [28] which involved coding transcripts in batches and meeting for calibration every ten transcripts. To maintain coding rigor, interrater reliability between coders was calculated and coding reconciled at multiple timepoints and cumulatively to ensure the intraclass coefficient was greater than 0.7 [29]. The study team then reviewed coding to develop themes. Themes related to AYACS’ financial challenges are presented in this paper. Quotes were edited minimally for clarity and brevity. Appendix 2 includes COREQ reporting guidelines and codebook relevant to financial challenges. Themes and quotes are described in the “Results” section, and data interpretation is described in the “Discussion” section.

### Mixed method integration

A convergent mixed-method approach was used to explore how a personal cancer diagnosis impacted financial well-being. Findings were integrated using merging, in which qualitative data were analyzed separately from quantitative data. Findings were then compared and contrasted by the research team in a joint display [30]. Thematic findings were also compared based on sex, diagnosis age, and treatment phase.

## Results

### Participant demographics

Seventy-six participants were approached and 41 enrolled (53.9%); six withdrew. Withdrawal reasons included logistics (i.e., participants were too busy to participate) and being lost to follow-up prior to the study visit. Thus, full data were collected from 35 participants (Table 1). Quotes were analyzed from 34 participants given one participant’s interview was unintentionally not audio-recorded due to technical difficulties; these 34 participants were included in mixed methods integration. Then, 51.4% were male, 82.8% were White, and 97.1% were non-Hispanic/Latino. Leukemias and lymphomas represented the greatest proportion of cancer diagnoses (74.3%). Most (62.9%) were posttreatment survivors, enrolled in school (60.0%), and employed part-time or full-time (57.1%). All participants self-reported being insured.

**Table 1:** Demographics of participant sample (*n* = 35)

**Age at interview (years)**	Mean (SD) or n (%)
Mean (SD)	22.5 (3.92)
Median	22.7
Range	16.4–29.1
**Participant identified sex**	
Female	17 (48.6%)
Male	18 (51.4%)
**Race**	
Asian/Pacific Islander	2 (5.7%)
Black/African American	2 (5.7%)
Hispanic/Latino	1 (2.9%)
Native American	1 (2.9%)
White	29 (82.8%)
**Ethnicity**	
Hispanic/Latino	1 (2.9%)
Not Hispanic/Latino	34 (97.1%)
**Age group and treatment phase** 15–21 years, receiving treatment 22–25 years, receiving treatment 15–21 years, completed treatment 22–25 years, completed treatment	10 (28.6%) 3 (8.6%) 12 (34.2%) 10 (28.6%)
**Cancer diagnosis** Acute lymphoblastic leukemia Acute promyelocytic leukemia Mixed phenotypic acute leukemia Chronic myeloid leukemia Hodgkin lymphoma* Peripheral T cell lymphoma Adrenocortical carcinoma Breast cancer Nasopharyngeal carcinoma Ewing sarcoma Soft tissue sarcoma (angiosarcoma) Juvenile pilocytic astrocytoma Malignant mixed germ cell tumor Testicular cancer	9 1 1 1 13 1 1 2 1 1 1 1 1 1
**Education** Enrolled in high school Enrolled in college Enrolled in technical/professional school Enrolled in graduate school Not enrolled—completed high school Not enrolled—completed some college Not enrolled—completed college Not enrolled—completed technical/professional school	8 (22.9%) 10 (28.6%) 1 (2.9%) 2 (5.7%) 4 (11.4%) 1 (2.9%) 8 (22.9%) 1 (2.9%)
**Employment**	
Not currently employed	15 (42.9%)
Currently employed full-time	12 (34.3%)
Currently employed part-time	8 (22.9%)
**Who do you live with? (check all that apply)** Significant other Siblings Parents Parents and significant other Parents and siblings Other (lives alone, declined response, and lives with other family members)	6 (17.1%) 3 (8.6%) 13 (37.1%) 2 (5.7%) 7 (20.0%) 4 (11.4%)
**Insurance status**	
Insured	35 (100%)

*One participant was diagnosed with Hodgkin lymphoma at age 24, and 2 years later was diagnosed with chronic myeloid leukemia

### Quantitative results

The mean COST score was 30.4 (SD 7.26), ranging from 16 to 43 (Table 2). No participants reported severe financial toxicity, with 77% with little to no financial toxicity (Table 3). No statistically significant differences in COST scores were noted when compared by sex, diagnosis age, or treatment phase, with COST analyzed as a continuous or categorical measure (Tables 2 and 3). However, we noted that 27.3% of those who completed treatment had moderate financial toxicity compared to 15.4% of those who were receiving treatment, and 38.5% of those age 22–25 had moderate financial toxicity compared to 13.6% of those age 15–21 (Table 3).

**Table 2:** Overall descriptive statistics and *T*-tests of COST scores

	Mean (SD)	Range	Mean Difference (95% CI)	*p-value*
**Overall sample** (*n* = 35)	30.4 (7.26)	16.0, 43.0	–-	–-
**Sex**				
Female (*n* = 17)	29.8	26.5, 33.1	−1.1 (−6.2, 3.9)	0.7
Male (*n* = 18)	30.9	26.9, 35.0		
**Age at diagnosis**				
15–21 (* n* = 22)	31.8	28.4, 35.2	3.7 (−1.4, 8.8)	0.1
22–25 ( *n*= 13)	28.1	24.4, 31.7		
**Treatment status**				
Receiving treatment (* n* = 13)	32.1	27.4, 36.8	−2.7 (−7.8, 2.5)	0.3
Completed treatment (*n* = 22)	29.4	26.3, 32.5

**Table 3:** Numbers and proportions based on COST reference ranges

	**No or low financial toxicity (26 +), * n* (%)**		**Moderate financial toxicity (14–25),* n* (%)**	***p*-value**
Overall	27 (77.1)	8 (22.9)		
Female	13 (76.5)	4 (23.5)		1
Male	14 (77.8)	4 (22.2)		
Age 15–21	19 (86.4)	3 (13.6)		
Age 22–25	8 (61.5)	5 (38.5)		0.12
Receiving treatment	11 (84.6)	2 (15.4)		
Completed treatment	16 (72.7)	6 (27.3)		0.7

*Note.* COST scores of 26 or higher indicate no or low financial toxicity and scores of 14–25 indicate moderate toxicity (see manuscript for citation). No participants in this sample scored below 14 (severe financial toxicity)

### Qualitative results

Four themes emerged:

- **Theme 1:** Financial situations related to cancer treatment led to varying levels of support and conversation within families.
  Younger participants described familial and philanthropic support as helping financial challenges: *“But my parents and family were… fortunate enough to, you know, provide me the… financial support to go through school and have a house that I would, you know, rent and things like that… so, you know, we were pretty fortunate to have that aspect of everything (Participant 11, 15–21 years old and completed treatment, Low toxicity* (*COST 30), Acute lymphoblastic leukemia).”* Younger participants also described not being aware of cancer-related financial challenges: *“I don’t know like, obviously, all of the financial stuff. But just somewhat like knowing just all everything [institutional philanthropic funding] paid for (Participant 21, 15–21 years old and completed treatment, Low toxicity (COST 28), Hodgkin lymphoma).”*
  While some older participants described financial support, others described being self-sufficient or even offering support to family while receiving treatment: *“… I was 100% dependent on me… and some of my family members that I’m actually here with actually depended on me when times that—when they needed stuff (Participant 23, 22–25 years old and receiving treatment, Low toxicity (COST 29), Hodgkin lymphoma).”*
- **Theme 2:** Financial strain was significant for participants of all ages and impacted future planning.
  Younger and older participants described how financial impacts of cancer affected them negatively. Participants described job lock (i.e., staying at current employment to retain healthcare benefits): *“I have a job right now bringing in money… and I have long-term disability insurance through that, so that if I were to get sick again, I would have at least some flow of income for—you know, I forget the terms of the policy, but for… a period of time after that. And If I were to stop that and go back to school, I would lose that insurance, and I would lose the income and be taking on debt. So, for me, it just doesn't really seem like a risk that I'm willing to take, so … I can’t say for sure I would have went back to school… I was kind of open about it, but right now… it certainly doesn’t make me want to go back (Participant 1, 22–25 years old and completed treatment, Low toxicity (COST 29), Acute lymphoblastic leukemia).”*
  Participants also described how financial strain impacted future planning, including fertility preservation: *“… the fertility treatment was the biggest… aspect for the finances because… we—we did the preservation, and… insurance doesn’t cover that, like, at all—like, any insurance… so that was, like, something that I know that we were really, really stressed-out about. And… the first time that we did it, it was not successful. So we had to do it again… and it's not like, oh, we already paid. We already have the medicine. Just go ahead. It’s just, like, a second round (Participant 29, 22–25 years old and receiving treatment, Moderate toxicity (COST 24), Breast cancer).”*
- **Theme 3:** Participants experienced gratitude for philanthropic funding, while experiences with insurance were mixed.
  Younger participants were eligible for institutional philanthropic funding if < 22 years old at time of cancer diagnosis. This funding helped many younger participants, as did other sources of philanthropic funding: *“Because they– we never saw a bill or anything. It was always just straight like funded to [institutional philanthropic funding]. They would take care of it, which was definitely really helpful for us, obviously… [institutional philanthropic funding] is definitely incredible for paying for all of that. And, yeah, they’re very awesome (Participant 21, 15–21 years old and completed treatment, Low toxicity (COST 28), Hodgkin lymphoma).”*
  Participants had varying opinions on insurance support. While some had less favorable views on insurance, others were grateful for insurance coverage: *“For me… I was lucky. I had a job that provided me with insurance that covered the vast majority of my bills. But… I still took a look to see just how large those bills were that even insurance was getting. And the amounts were staggering… meaning that without my insurance, I would have had no help, clearing out all my funds, and there was—severely hindering whatever my family may have (Participant 30, 22–25 years old and completed treatment, Low toxicity (COST 28), testicular cancer).”*
- **Theme 4:** Due to the financial strain, some participants experienced stressful emotions (such as guilt and worry), while others did not.
  Older and younger participants described guilt for financial repercussions of cancer treatment on loved ones: *“And, like, this just—I felt bad because this was my burden, I guess (Participant 12, 15–21 years old and receiving treatment, Low toxicity (COST 26), Hodgkin lymphoma).”*Another described the personal financial burden: *“But there was no finan—there was no financial stress towards anybody besides myself (Participant 23, 22–25 years old and receiving treatment, Low toxicity (COST 29), Hodgkin lymphoma).”* Others, mainly younger AYACS, described thinking about professional goals due to less worry about their financial situation: *“And what’s nice about my situation is that, um, it’s—I don’t have to get a job for college… Like, everything is paid for. I don’t need to worry about it. I’m—just need to worry about education. That’s all.” (Participant 41, 15–21 years old and receiving treatment, Low toxicity (COST 43), acute lymphoblastic leukemia).*
  Some ascribed less financial strain due to their social support: *“It was good to have the support system and not having to worry about rent or bills (Participant 13, 15–21 years old and completed treatment, Low toxicity (COST 37), Hodgkin lymphoma).”*

#### Mixed method integration and joint display

To integrate findings, a joint display merging qualitative and quantitative results was constructed (Table 4). Interpretations and conclusions were iterated among the research team.

**Table 4:** Joint display

Qualitative	Quantitative	Interpretation
**Theme 1:** Financial situations due to cancer treatment led to various levels of support and conversations within AYACS’ families. - Younger participants often described familial help, and support from local philanthropic organizations - Younger participants also described not always being aware of, or being sheltered from the financial challenges of cancer treatment - While some older participants described familial financial support, others described being more self-sufficient, or still offering support to their family amidst treatment **-** Younger participants also described how they did not talk about financial aspects of their treatment with their family	- No significant differences in COST scores by sex, age at diagnosis, or treatment phase - A larger proportion of those who completed cancer treatment experienced moderate financial toxicity compared to those currently receiving cancer treatment (not statistically significant) - Higher proportions of those age 22–25 at time of cancer diagnosis experienced moderate financial toxicity, compared to the proportion of those who experienced moderate financial toxicity age 15–21 at time of cancer diagnosis (not statistically significant)	- Qualitative data and quantitative data were divergent in that more nuanced opinions on financial toxicity and stress were captured through qualitative data insofar as differences by diagnosis age and the role of social support in financial toxicity - Less financial worry by younger AYACS could lead to greater focus on future professional goals and achievement (and developmental stage) - Impact of financial toxicity not just during but after cancer therapy complete is important to consider - Financial toxicity impacts professional achievement - Support needed for AYACS (more philanthropic money for pediatric patients)
**Theme 2:** Financial strain was significant for participants of all ages and impacted future planning for: - Education - Work (including job lock) - Medication adherence - Fertility preservation		
**Theme 3:** Participants experienced gratitude for philanthropic funding, while experiences with insurance were mixed. - Differing opinions on insurance - Various sources of philanthropic funding helped participants - Internal philanthropic support a significant source of financial support for younger participants		
**Theme 4:** Due to financial strain, some participants experienced stressful emotions, while others did not. -Younger and older participants described feelings of guilt for the financial repercussions of their cancer treatment on those that care for them. This sentiment occurred even for those in the midst of cancer treatment -Mainly younger AYACS described being able to think about future professional and academic goals and other endeavors, due to less worry about their financial situation		

## Discussion

This mixed methods study builds on literature about AYACS’ financial challenges by revealing differences in financial experiences, especially between younger and older AYACS. While there were no statistically significant differences in COST scores by sex, diagnosis age, or treatment phase, our qualitative findings revealed differing experiences based on diagnosis age. Firstly, younger AYACS described help from loved ones and institutional philanthropic support as mitigating financial stress, whereas older participants did not always have access to these resources. Secondly, younger AYACS described thinking about future endeavors due to less worry or knowledge about familial financial situations. Thirdly, there is a need for more sensitive instruments to assess AYACS’ financial toxicity. Participants of all ages felt the impact of cancer’s financial consequences acutely and long-term and were grateful for opportunities to defray cancer care costs.

### Younger AYACS have greater financial support than older AYACS.

Younger AYACS described greater financial support than older AYACS, likely related to our institution offering significant direct (i.e., drug cost coverage) and indirect financial support (i.e., supportive care services) to those diagnosed with cancer if < 22 years old. Having this support may have enabled younger AYACS to think about their future without financial constraints impacting their envisioned trajectories. Another explanation could be that younger AYACS were developmentally unable to think about financial impacts of cancer given dependence on caretakers, while older AYACS’ financial stressors were more likely their personal burden. The age-related differences may also be because of younger AYACS’ lack of awareness of financial cancer-related conversations. Not having knowledge of finances could have been initiated from participants’ parents to protect participants, or from participants themselves. This sentiment of being protected from financial stresses was not prominent in older AYACS’ comments.

Our findings of older AYACS’ financial toxicity corroborate literature [18]. In one study, nearly 54% of AYACS 18–39 years old had COST scores < 26 (moderate/severe financial toxicity), with increased financial toxicity significantly associated with reduced quality of life, using more savings to pay for treatment, and taking less medication [31]. This difference may grow as many AYACS benefited from cancer treatment coverage due to the Affordable Care Act. This federal plan requires issuers to offer dependent child coverage until the adult child reaches 26 years old [32]. Overturning of this law could impact even more AYACS and insurance coverage options.

Even without our institutional financial support, many pediatric cancer centers offer services to defray cancer treatment costs. While less than 10% of the federal budget is spent on pediatric cancer [33], pediatric cancer is one of the highest funded cancer groups insofar as nonprofit organization funding (which often funds supportive cancer care including infrastructure grants and supportive care programming) [34]. A pediatric oncology site is where younger AYACS often receive care and can access this funding. AYA-prevalent cancers treated in adult centers like breast cancer, leukemia and lymphomas were also well-funded in proportion to their incidences. However, AYA-prevalent cancers like gastrointestinal (including colorectal), gynecologic, and lung cancers were poorly funded in proportion to their incidences [34]. While not directly applicable to our sample, this funding disparity could explain differences in supportive care access for older AYACS. Moreover, the supportive care services in pediatric cancer centers can lend additional social [35] and psychological [36] support to AYACs.

### Younger AYACS think about their future with less worry than older AYACS, impacting downstream goals and health.

Despite the importance of professional achievement for AYACS [37], this study revealed how younger AYASs could think about future plans due to less worry or knowledge about familial financial situations, compared to older AYACS. This is not surprising given the strong association of cancer-related financial toxicity as a barrier to professional advancement [38]. Many AYACS become derailed from professional advancement [38–40] and are less likely to complete school, with detrimental downstream effects in workforce productivity [41] and decreased earnings [42]. As most AYACS are cured of cancer, they accumulate financial complications related to cancer, with potentially greater risk for bankruptcy compared to those without cancer [43].

Among AYACS, worse financial toxicity is significantly associated with higher levels of depressive and anxiety symptoms and lower self-efficacy [14]. In turn, psychological health costs can increase financial toxicity among AYACS [15]. By equipping AYACS with resources to decrease cancer’s financial burden, we may be able to change the trajectory of their professional achievement and health.

### There is a need for more sensitive financial toxicity measures for AYACS.

While there were no statistically significant differences in financial toxicity across several demographic factors, our qualitative findings revealed differences by diagnosis age which warrant further investigation. Interestingly, while our qualitative themes align well with COST constructs (e.g., financial toxicity’s impact on emotional health, professional achievement, and future finances), one unique domain in our qualitative data was the protectiveness of social support on financial toxicity. Social support (in the form of family, friends, and healthcare personnel) may have alleviated financial toxicity for younger AYACS, and may also partly explain why those who completed treatment may experience continued financial toxicity (i.e., less frequent cancer care and decreased perceived need for support by others). COST does not capture social dimensions as much as previously mentioned constructs. Our qualitative findings suggest additional questions related to social supports/social networks could be a helpful inclusion in future quantitative financial toxicity measures for AYACS.

### Study strengths and limitations

Strengths included the mixed-method study design, which allowed us to capture qualitative data to more comprehensively understand financial toxicity among AYASs. We collected data from AYACs in various treatment phases and diagnoses. We had robust male representation (important as males are underrepresented in AYACS psychosocial studies [44]) and a high proportion of patients with leukemias and lymphomas, representative of the significant proportion of these malignancies among AYACS [45]. About two-thirds of our participants were posttreatment survivors, which provides a rich perspective from those who could reflect on their experiences.

We justified the use of COST as the current age for most of our participants (~ 80%) was ≥ 18; thus a minority (~ 20%) was < 18 years old. Furthermore, there is no validated measure that can be used universally within AYACS. Other limitations included being a single-center study with a majority of White/Non-Hispanic participants, so results may not be generalizable to areas that are demographically different. Our participants’ COST scores were higher with no participants with severe financial toxicity, possibly due to being insured by self-report and having access to philanthropic support, which could influence data interpretations in different settings. Importantly, our institution benefits from philanthropic funding for those diagnosed with cancer < 22 years old, which could have disproportionately decreased financial challenges of younger compared to older AYACS. Our study focused on younger AYACS treated at an academic center, so results may not generalize to older AYACS 26–39 years old or those treated in community settings. Additionally, as most participants were diagnosed with hematologic malignancies, our study may be insufficient to identify financial toxicity with other cancer types. Our qualitative results from post-treatment survivors may have conflated both current and past financial challenges. Our study excluded participants who were non-English speaking, with relapsed cancer, or with cognitive or physical inability to participate, which may have skewed our findings toward those with less financial challenges and less financial toxicity than other AYACS. Our quantitative analyses were likely underpowered to detect differences given our small sample; thus, our quantitative results are more descriptive or exploratory in nature. Lastly, this was a small sample which could affect generalizability.

In conclusion, AYACS across sex, age, and treatment phase experienced financial challenges. Our qualitative data uncovered important differences in experiences of younger vs. older AYACS, particularly related to social support in protecting against financial toxicity. This study provides direction to improve resources to decrease financial toxicity for all AYACS, and design more sensitive quantitative instruments to assess financial toxicity among AYACS with additional focus on social constructs of financial support.

## Supplementary Information

Below is the link to the electronic supplementary material.

ESM 1 (DOCX 59.2 KB)

## Data Availability

The data generated during and/or analyzed during the current study are not publicly available, nor are they available on request due to preserving participant anonymity.
