# Supplementary material for: The impact of financial toxicity on adolescent/young adult cancer survivors (AYACS): A mixed methods study
Source: Support Care Cancer. 2026 Sep 2;34(9):921. doi: 10.1007/s00520-026-11093-z (PMC13538235; doi:10.1007/s00520-026-11093-z)
Supplement: Supplementary file 1 [file 520_2026_11093_MOESM1_ESM.docx]

**Appendix 1: Interview Guide**

**Aim 1: Qualitatively explore the psychosocial challenges and resource utilization of AYA cancer**

**survivors as they relate to anxiety, depression, financial toxicity**

- OPENING: We know that in addition to the medical issues that go with being diagnosed and

treated for cancer, many patients your age experience a great deal of other challenges both during

cancer treatment and after. How has your experience with cancer treatment affected you?

- PROMPTS IF NEEDED:

• Have you experienced any psychological challenges? If yes, tell me more about that.

• Have you ever experienced any challenges related to mental health? If so, can you

tell us about that?

• Have you ever received any formal mental health diagnoses? Tell me more about

that.

- Many patients your age say that their relationships with family, friends or others in their lives changed after they were diagnosed with cancer. Did having cancer change your relationships with family, friends or others in your life? How or how not?
- Did any of your healthcare providers ever ask you how your relationships with family, friends or others in your life were going during cancer treatment, or after cancer treatment was finished? If so, do you remember who asked about that?
- Do you feel that connecting with other people your age who have also gone through cancer treatment would be helpful or an additional support during cancer treatment or after cancer treatment? Why or why not?
- Many patients use different services such as a social worker, psychologist, or chaplain during

cancer treatment and after. Have you ever used these services? What was your experience using

these services?

- Are there any services that you wish you had available to you that were not offered to you?

Explain.

- We are interested in learning more about how cancer treatment affects young patients and

families with regards to finances. Is this something that affects you? How so?

- PROMPTS IF NEEDED:

• How, if at all, has your cancer impacted yours or your family’s finances?

• Have financial issues been something you and your family have talked about?

• What services, if any, have you received during cancer therapy or after to help

reduce financial burdens?

**Aim 2: Qualitatively explore how AYA cancer survivors’ experiences with cancer treatment**

**affect educational and occupational aspirations**

- OPENING: We know that many patients your age say that school can be challenging to do during

and after cancer treatment. How, if at all, has your cancer treatment affected your educational

goals?

- PROMPTS IF NEEDED:

• Tell me about any goals for school you had before the diagnosis of cancer?

• Did these goals for school change while you were being treated for cancer, or after?

If yes, how so?

• Did going through cancer treatment change what you wanted to do as far as

educational goals?

• How do you feel about going back to school?

• Do you feel supported in your return back to school after cancer treatment? If yes,

how so?

• For those who have completed treatment:

- After you finished cancer treatment, did you go to school? How soon

after? How was the process to integrate back to school? Is there

anything anyone could have done to make the process easier?

- OPENING: We know that many patients your age say that having a job can be challenging to do

during and after cancer treatment. How, if at all, has your cancer treatment affected your

occupational goals?

- PROMPTS IF NEEDED:

• Tell me about any goals for work you had before the diagnosis of cancer?

• Did these goals for work change while you were being treated for cancer, or after? If

yes, how so?

• Did going through cancer treatment change what you wanted to do as far as your

career?

• How do you feel about going back to work?

• Do you feel supported in your return back to work after cancer treatment? If yes,

how so?

• For those who have completed treatment:

- After you finished cancer treatment, did you go to work? How soon

after? How was the process to integrate back to work? Is there

anything anyone could have done to make the process easier?

**Appendix 2: COREQ Reporting Guidelines and Codebook**

**Domain 1: Research Team and Reflexivity**

*Personal characteristics:* Staff 1 is a research technologist with a B.S. in Biology and Psychology with a career interest in research dealing with behavioral psychology. Under the guidance of Staff 2, she conducted interviews for this study, with an understanding of the importance of research objectivity. Like Staff 2 she has prior experience conducting interviews of AYACS. Together with Staff 2 she coded and participated in theming of this study.

Staff 2 has multiple years of experience in qualitative and mixed-methods research and project management. Given her ample experience in conducting interviews and focus groups, she realizes the importance of objectivity during design and analysis as a result of possible bias with connections to the topic. She has prior experience working with the adolescent/young adult cancer survivor (AYACS) patient population and understands the sensitivity of the topics discussed in these interviews. She led the coding and theming of this study together with Staff 1.

The lead qualitative researcher (Staff 3) is an established physician scientist whose research interest focus on end of life and communication issues. Her qualitative work generally follows a pragmatic approach using descriptive methods to understand phenomena, and she applies a humanistic perspective to data analysis. She oversaw data theming and analysis.

Staff 4, a clinician-scientist, is the principal investigator of this project. Her research interests, largely informed by the clinical care she provides to AYACS, focuses on better understanding of the psychosocial issues of AYACS to devise effective interventions to improve long-term goal achievement and health-related behaviors of this patient population. Keenly aware of her potential for bias, she purposely did not participate in interview conduct for this study, nor the coding. She participated in theming for this study together with Staff 1 and Staff 2 who were closest to the data. Staff 1, 2, 3 and 4 all concurred on the final themes for this manuscript.

(i) *Relationship with participants:* As Staff 4 either provides clinical care for or knows many of the AYACS in this study, she proactively did not conduct interviews or code the qualitative data, in order to maintain data objectivity. For this reason, Staff 1 and Staff 2 conducted all of the interviews and coding. Staff 1 and Staff 2 have prior experience performing interviews with this patient population.

**Domain 2: Study Design**

(i) *Theoretical framework:* Thematic analysis was used to analyze the data. We used a phenomenologic approach that explored the phenomenon of financial toxicity and how a personal cancer diagnosis impacted AYACS’ experiences related to financial well-being. This approach avoids using preconceived categories and is appropriate to use when literature on a topic is limited.
(ii) *Participant selection:* Study participants were AYACS who were ages 15-25 years old at the time of cancer diagnosis and still within 6 years of their initial cancer diagnosis. Data collection occurred from the Winter of 2021 to the Summer of 2022. Recruitment was conducted remotely until early 2022 when COVID-19 precautions were lifted. This younger AYACS subset was chosen to study those undergoing similar developmental and interpersonal challenges to each other (e.g., developing autonomy and personal values/identity, cultivating peer relationships, preparing to enter the workforce). Participants were receiving or had received oncologic care at a tertiary care center that serves both urban and rural counties. Participants were recruited through review of the institutional cancer registry, the divisional cancer database, the electronic medical record, and clinic templates of providers. Additional inclusion criteria for participants (and consenting parents of participants < 18 years old) included being fluent in written and spoken English and having access to a computer or smartphone. Participants who were non-English speaking were excluded due to available study team resources. Participants with relapsed cancer or with cognitive or physical inability to participate were excluded to ensure these characteristics would not confound exploration of factors impacting psychological health.
(iii) *Setting:* After in-person recruitment, the study team and participant set up a mutual time over the videoconferencing platform Zoom to perform the study assessment. All data collection was conducted over Zoom.
(iv) *Data collection:* Two trained qualitative interviewers (Staff 1 and Staff 2) utilized a semi-structured interview guide to conduct one-on-one interviews with participants via videoconference. Interviews lasted approximately 30-45 minutes, were audio-recorded, and transcribed verbatim by a professional transcription service. The interview guide broadly explored several topics. The qualitative data related to financial toxicity is presented in this paper.

**Domain 3: Analysis and Findings**

(i) *Data analysis:* Thematic analysis was used to analyze data using a phenomenological approach to explore AYACS’ experiences related to financial consequences after a personal cancer diagnosis. A phenomenological approach seeks to understand a phenomenon by exploring it from the perspective of those who experienced it and avoids using preconceived categories; it is appropriate to use when literature on a topic is limited. MAXQDA software was used to organized and manage data. Research team members with qualitative research experience reviewed approximately 10-20% of the total dataset to inductively create preliminary categories, codes, and sub-codes describing the data. A preliminary codebook for ‘Finances’ and its related codes was constructed and used to code five transcripts after which discrepant codes were adjudicated by group discussion to create a final codebook with codes, definitions, and exemplars. Two coders then coded the entire dataset using the constant comparison method which involved coding transcripts in batches and meeting for calibration every ten transcripts. To maintain coding rigor, inter-rater reliability between coders was calculated and coding reconciled at multiple timepoints and cumulatively to ensure the intraclass coefficient was greater than 0.7. The study team then reviewed coding to develop themes. Themes related to AYACS’ financial challenges are presented in this paper. Quotes were edited minimally for clarity and brevity.
(ii) *Reporting:* The manuscript discusses the themes that arose from coding and theming. Supporting participant quotes are provided. Quotes were minimally edited for clarity when appropriate.

**Financial Toxicity Codebook**

| Finances | Subcode | Definition | Example quote |
| --- | --- | --- | --- |
|  | 1Ai. Impacts on personal finance | Mentions of impact on personal finance | Like my mother or my sister or, you know, just anybody that needed help, I would help out. I would be that person in the family to help out. (Participant 23) |
|  | 1Aii. Impacts on family finance | Mentions of impact on family finance | Her periods where, mom was not working, and now she is, and t think that has been helpful, but… You know, it-- it's just obviously an added, like, monthly expense, if you will. Like it's-- it's something… that's pretty consistent, so I-- I know that was-- I don't know the-- I don't know to what extent it was challenging, but I know at times that that had to be challenging… to have that added to other normal expenses that you would have. (Participant 1)  But I would imagine that the amount of pills and stuff that I've taken is not a small amount of money worth. OK. So I would imagine that takes a lot of stress off my parents. But for me, personally, I really have no kind of concept of that at all because I've never really had to pay a medical bill, right? Because I'm-- I'm just too young to pay for it, you know. (Participant 22) |

**Appendix 3: Themes and Supporting Participant Quotes**

| **Themes** | **Supporting Quotations** |
| --- | --- |
| **Theme 1: Financial situations related to cancer treatment led to varying levels of support and conversation within families.** | |
| **Subtheme 1a:**  Some younger participants experienced financial support through monetary value (e.g., jobs, philanthropic funding, family members) and through family conversations about ways to cover the cost of cancer treatment. | “… it was pretty good…. A little bit strained… and my mom worked at, like, a farmer's market. They manage.” (Participant 12, 15-21 years old and receiving treatment, Low toxicity (COST 26), Hodgkin lymphoma)  “I don't know. But I think [my mother] would have made it work either way… But I know my mom and dad were like we can make it work.” (Participant 13, 15-21 years old and completed treatment, Low toxicity (COST 37), Hodgkin lymphoma)  “Because they-- we never saw a bill or anything. It was always just straight like funded to [institutional philanthropic funding]. They would take care of it, which was definitely really helpful for us, obviously… [institutional philanthropic funding] is definitely incredible for paying for all of that. And, yeah, they're very awesome.” (Participant 21, 15-21 years old and completed treatment, Low toxicity (COST 28), Hodgkin lymphoma)  “… because of the [institutional philanthropic funding] and all that they-- it didn't financially impact them really at all, other than me convincing… my dad to get me a dog because it was going to make me feel better.” (Participant 19, 15-21 years old and completed treatment, Moderate toxicity (COST 20), Acute lymphoblastic leukemia)  “But just, kind of, sitting down and, like, looking at the bills with everything, and both my parents were like, we don't care how much it's going to cost as long as you get better. We're going to find a way to, you know, pay for all this no matter what the cost is… but like I said, we were just very fortunate to have that health insurance and both of my parents working full time positions and things like that.” (Participant 11, 15-21 years old and completed treatment, Low toxicity (COST 30), Acute lymphoblastic leukemia)  “And, you know, in today's world, houses are crazy. So how do you afford a house and afford a car, and afford everything you need? And that's enough, and then you throw this on it, it definitely stresses me out. But like, as far as my family, my family did really well. Like we had-- my job was very good with their-- they actually covered, or kept paying me, when I was off. So that was really nice. They still gave me my paycheck every week, and even though I told them they don't have to.” (Participant 4, 15-21 years old and receiving treatment, Moderate toxicity (COST 17), Chronic myeloid leukemia)  “I don't know like, obviously, all of the financial stuff. But just somewhat like knowing just all everything [institutional philanthropic funding] paid for.” (Participant 21, 15-21 years old and completed treatment, Low toxicity (COST 28), Hodgkin lymphoma)  “[when asked whether they discussed finances of cancer with their parents] Yeah, I have… it's not as much of a-- a topic anymore. But definitely, like, during treatment, it was something that we discussed.” (Participant 40, 15-21 years old and completed treatment, Moderate toxicity (COST 16), Hodgkin lymphoma)  “But my parents and family were… fortunate enough to, you know, provide me the… financial support to go through school and have a house that I would, you know, rent and things like that… so, you know, we were pretty fortunate to have that aspect of everything.” (Participant 11, 15-21 years old and completed treatment, Low toxicity (COST 30), Acute lymphoblastic leukemia)  “I'm trying to think-- in terms of like programs, I mean, I think-- because I did, like, have conversations, and have had recently conversations kind of about how they did it financially. I think it was mostly, like, from money they had from savings or, like, income from their job, so I don't think they used a lot of programs.” (Participant 1, 22-25 years old and completed treatment, Low toxicity (COST 29), Acute lymphoblastic leukemia) |
| **Subtheme 1b:**  Older participants experienced varying levels of financial support, and younger participants had various reasons for not conversing about their cancer treatment-related financial situation. | “Like my mother or my sister or, you know, just anybody that needed help, I would help out. I would be that person in the family to help out.” (Participant 23, 22-25 years old and receiving treatment, Low toxicity (COST 29), Hodgkin lymphoma)  “Great, it was definitely challenging. There is a lot of, like, costs that we had to do. Like, obviously, my family was showing up at the hospital every week, every single day, and that was, like, an hour drive just one way. So a lot of expenses for gas, and travel, and then also buying food while they were up here.” (Participant 32, 22-25 years old and completed treatment, Moderate toxicity (COST 25), Mixed phenotypic acute leukemia)  “… my mother was assisting me financially. But at the same time, she was doing financially poor, so she couldn't support me very much.” (Participant 34, 22-25 years old and completed treatment, Moderate toxicity (COST 22), Peripheral T cell lymphoma)  “So after my father passed, he was like the main breadwinner of our family. So my mom works part-time, doesn't make much. And my brothers and I all chip in to help pay for the house that my mom and my one brother and I are living in now. So we all chip in to pay for this place… and so, that plays a part in it too, I guess, just making ends meet for not only me, but like this household and… my family as a whole.” (Participant 36, 22-25 years old and completed treatment, Moderate toxicity (COST 25), Hodgkin lymphoma)  “… I got a lot of great support through being treated in the Children's Hospital. But… you know, not being on the [institutional philanthropic funding], that the bills after insurance were on my parents.” (Participant 1, 22-25 years old and completed treatment, Low toxicity (COST 29), Acute lymphoblastic leukemia)  “So I have never been the money maker in our family, or the breadwinner, you could say… I went to school for early education. And when I was… diagnosed the second time… I was working as a social worker with some foster kids. So that not very lucrative…But between what my husband makes and what… the insurance picked up… we-- we didn't get hurt as much as some other people probably did.” (Participant 38, 22-25 years old and completed treatment, Low toxicity (COST 33), Hodgkin lymphoma)  “Ah, my family helped [financially].” (Participant 10, 22-25 years old and completed treatment, Moderate toxicity (COST 16), Breast cancer)  “… I was 100% dependent on me… and some of my family members that I'm actually here with actually depended on me when times that-- when they needed stuff.” (Participant 23, 22-25 years old and receiving treatment, Low toxicity (COST 29), Hodgkin lymphoma)  “But I do have, like, my family that supports me, and they will always be [with me] in case anything.” (Participant 32, 22-25 years old and completed treatment, Moderate toxicity (COST 25), Mixed phenotypic acute leukemia)  “So-- but I-- I never asked a lot. But then, after like-- it took a while for… the government to… start supporting me. But then… when they did actually start supporting me, I felt like, you know-- I felt like I no longer needed my mother to support me.” (Participant 34, 22-25 years old and completed treatment, Moderate toxicity (COST 22), Peripheral T cell lymphoma) |
|  | “But… it shed more light on, like, the fact that I didn't look at, like, the financials, I guess. That wasn't really my area of expertise.” (Participant 12, 15-21 years old and receiving treatment, Low toxicity (COST 26), Hodgkin lymphoma)  “I don't think she would have really made it transparent how down under we would land, but I think I would have known. I had the knowledge for these treatments, names and anything out of pocket is going to be really expensive.” (Participant 13, 15-21 years old and completed treatment, Low toxicity (COST 37), Hodgkin lymphoma)  “[when asked if they discussed cancer treatment cost with family] We didn't actually.” (Participant 15, 15-21 years old and completed treatment, Low toxicity (COST 36), Hodgkin lymphoma)  “[when asked if they discussed cancer treatment cost with family] Just never talked about it.” (Participant 17, 15-21 years old and receiving treatment, Low toxicity (COST 32), Nasopharyngeal carcinoma)  “No… I mean, maybe out here like a thing here or there, like this would be really expensive, but it's not because we get this-- all this charity, you know. I hear that every once in a while. But it's not like something we talk about because that's kind of like walking on eggshells thing. And I don't want to talk about anything associated with my treatment at all, so I wouldn't want to talk about the money behind it either, especially if I have nothing to do with the money behind it.” (Participant 22, 15-21 years old and receiving treatment, Low toxicity (COST 41), Acute lymphoblastic leukemia)  “I wasn't too worried about it, though, because my family doesn't talk much finances with me. But I'm pretty sure that even if we didn't have [institutional philanthropic funding], we would have been all right paying for all the medical stuff.” (Participant 39, 15-21 years old and completed treatment, Low toxicity (COST 34), Malignant mixed germ cell tumor (retroperitoneum))  “I haven't really talked to my parents about what they had [to] pay out of pocket.” (Participant 37, 15-21 years old and completed treatment, Low toxicity (COST 36), Ewing sarcoma) |
| **Theme 2: Financial strain was significant for participants of all ages and impacted future planning.** | |
| **Subtheme 2a:** Participants struggled with financial challenges of treatment. | “But just being without income for so long, like, I had a decent amount of savings and it pretty much completely… wiped it all out… it also, you know, drained a lot from my family, too, who were able to help with that… there's still bills that we're, like, working to pay off that just take time because of how big they are... so it was definitely negatively impacted by that pretty significantly…” (Participant 40, 15-21 years old and completed treatment, Moderate toxicity (COST 16), Hodgkin lymphoma)  “FMLA is a very annoying system.” (Participant 30, 22-25 years old and completed treatment, Low toxicity (COST 28), Testicular cancer)  “I would say it was unfortunate watching my savings deplete, especially just graduating college. Because I sort of had something, but now, I don't really have anything anymore.” (Participant 32, 22-25 years old and completed treatment, Moderate toxicity (COST 25), Mixed phenotypic acute leukemia)  “So financially, by myself, I'm not that strong right now. You know, I had a good bit of money saved up beforehand.” (Participant 3, 15-21 years old and receiving treatment, Low toxicity (COST 26), Acute lymphoblastic leukemia)  “Well, it really… I've had a lot of, like, financial problems… It definitely impacted me like with my… medical assistance stopped paying for a lot of my stuff.” (Participant 10, 22-25 years old and completed treatment, Moderate toxicity (COST 16), Breast cancer) |
| **Subtheme 2b:** Several participants reported financial difficulty due to cancer and its treatment. | “So I knew I couldn't work and I had bills, car insurance, and stuff like that, so a lot of it [my mother] would help there.” (Participant 13, 15-21 years old and completed treatment, Low toxicity (COST 37), Hodgkin lymphoma)  “I think the-- the biggest problem was from… probably from missing work.” (Participant 3, 15-21 years old and receiving treatment, Low toxicity (COST 26), Acute lymphoblastic leukemia)  “I was still working. I was not getting… I was obviously not there all the time, so I was not getting my salary.” (Participant 27, 22-25 years old and completed treatment, Low toxicity (COST 31), Hodgkin lymphoma)  “And… yeah, it took a while. I don't really know how long I've been in that stage of, like, doing maintenance chemo, getting, like, government assistance-- financial assistance, and not working. It, like-- it was, like, quite a while.” (Participant 34, 22-25 years old and completed treatment, Moderate toxicity (COST 22), Peripheral T cell lymphoma)  “But apart from that, like going through cancer, I-- I wasn't working. So I didn't have any sort of income at all.” (Participant 36, 22-25 years old and completed treatment, Moderate toxicity (COST 25), Hodgkin lymphoma)  “Well, my personal finances, obviously I couldn't work. So that's the only way it impacted me.” (Participant 39, 15-21 years old and completed treatment, Low toxicity (COST 34), Malignant mixed germ cell tumor (retroperitoneum))  “… so that-- that was the main thing, like having some kind of, like, more wide financial support would have been nice, because I-- I really had no income, basically, for that entire time… I really had no capacity to work.” (Participant 40, 15-21 years old and completed treatment, Moderate toxicity (COST 16), Hodgkin lymphoma)  “Because I had to like quit my job, but then I was like still on my parents' insurance. But then I eventually had to switch to like Medicaid. And then because I was disabled for a period of time, it was like Medicare and all different things… it is very confusing especially when you're very ill to…try to navigate that.” (Participant 5, 22-25 years old and completed treatment, Low toxicity (COST 31), Acute lymphoblastic leukemia)  “And then… you know, I wasn't able to work… and then… whenever I did finish treatment, I had gotten, you know, I-- I kind of, strictly want to stay with the part time jobs.” (Participant 11, 15-21 years old and completed treatment, Low toxicity (COST 30), Acute lymphoblastic leukemia)  “But, you know… I couldn't work and make money and, you know, get hours that way, so that was definitely different.” (Participant 9, 15-21 years old and completed treatment, Low toxicity (COST 30), Acute lymphoblastic leukemia) |
| **Subtheme 2c:** Some participants changed their actions during and after cancer treatment, partly related to their financial situation. | “So I just didn't want to spend that money. And especially when I can just stay at home in my own room and have my mom's home-cooked food and not have school cafeteria food, that's a steal for me.” (Participant 13, 15-21 years old and completed treatment, Low toxicity (COST 37), Hodgkin lymphoma)  “You know, just basically-- just hustling out there, and, you know, trying to build my life… And I was actually… what's the word? Able to… provide for myself this whole time. So even if that means I have to do less stuff or, you know, save more money and just spend money whenever I need to spend money instead of want to spend money, I was able to, you know, provide for myself and do that.” (Participant 23, 22-25 years old and receiving treatment, Low toxicity (COST 29), Hodgkin lymphoma)  “Because I just-- I just-- I know the value of, like, having a good roof over my head.” (Participant 34, 22-25 years old and completed treatment, Moderate toxicity (COST 22), Peripheral T cell lymphoma)  “It kind of made me realize, like, how big it actually is-- not even the actual price of chemotherapy meds, but even just the small pills I was getting in the mornings. I would look up the price and be just amazed. Like, wow, this pill costs a decent amount of money. I better be eating this. I don't care if I'm nauseous or not.” (Participant 37, 15-21 years old and completed treatment, Low toxicity (COST 36), Ewing sarcoma)  “… the fertility treatment was the biggest… aspect for the finances because… we-- we did the preservation, and… insurance doesn't cover that, like, at all-- like, any insurance… so that was, like, something that I know that we were really, really stressed-out about. And… the first time that we did it, it was not successful. So we had to do it again… and it's not like, oh, we already paid. We already have the medicine. Just go ahead. It's just, like, a second round.” (Participant 29, 22-25 years old and receiving treatment, Moderate toxicity (COST 24), Breast cancer) |
| **Subtheme 2d:** The financial burden from cancer treatment led some participants to reconsider future plans. | “It's weird, 'cause it's like, it-- yeah, it feels manageable, but it's like, I know as soon as I get better, I'm probably going-- I'mma still be doing what I need to do. Like, how I do it now, from home. But I'mma still be doing those other things, too, so it might be, you know, 18 hour [work] days again, just because I enjoy doing that, because I'm growing right now. Don't have any children, trying to make sure my life is set--for when I have the children and have a wife, you know? I could be able to provide for them and anything they would need.” (Participant 23, 22-25 years old and receiving treatment, Low toxicity (COST 29), Hodgkin lymphoma)  “I think the only thing was, they were worried about how that affected ability to pay for college. But with, again, with [institutional philanthropic funding] entering the… equation, that stress was, kind of, minimized.” (Participant 7, 15-21 years old and completed treatment, Low toxicity (COST 31), Juvenile pilocytic astrocytoma)  “And maybe pick up a part-time job around here in order to get more money, but other than that, it wouldn't-- it's not too far out of the range. It's not like I'm saving up for a brand new car or anything, you know. It's an investment that I would have to work towards to be able to go overseas. But it wouldn't be so far out there that I couldn't feasibly work towards.” (Participant 22, 15-21 years old and receiving treatment, Low toxicity (COST 41), Acute lymphoblastic leukemia)  “[I do worry] A little bit, if I was to decide to go try something else [an alternate treatment not covered by insurance].” (Participant 6, 15-21 years old and receiving treatment, Low toxicity (COST 35), Adrenocortical carcinoma)  “I have a job right now bringing in money… and I have long-term disability insurance through that, so that if I were to get sick again, I would have at least some flow of income for-- you know, I forget the terms of the policy, but for… a period of time after that. And If I were to stop that and go back to school, I would lose that insurance, and I would lose the income and be taking on debt. So, for me, it just doesn't really seem like a risk that I'm willing to take, so … I can't say for sure I would have went back to school… I was kind of open about it, but right now… it certainly doesn't make me want to go back.” (Participant 1, 22-25 years old and completed treatment, Low toxicity (COST 29), Acute lymphoblastic leukemia) |
| **Theme 3: Participants experienced gratitude for philanthropic funding, while experiences with insurance were mixed.** | |
| **Subtheme 3a:** Younger participants cited institutional philanthropic funding as a positive influence on their financial situation throughout their cancer journey. | “[Institutional philanthropic funding] would help because [insurance] would just decline things for no reason.” (Participant 13, 15-21 years old and completed treatment, Low toxicity (COST 37), Hodgkin lymphoma)  “They did-- they were giving us meal vouchers and… gas vouchers. Those were definitely, like, very helpful, and… just let us have extra money to put towards, like, other things for the house and stuff. And we didn't have to worry while I'm in the hospital, that, you know, we weren't going to be able-- we'd have to spend extra money on the food and getting there, with the gas [and] stuff.” (Participant 17, 15-21 years old and receiving treatment, Low toxicity (COST 32), Nasopharyngeal carcinoma)  “Because they-- we never saw a bill or anything. It was always just straight like funded to [institutional philanthropic funding]. They would take care of it, which was definitely really helpful for us, obviously… [institutional philanthropic funding] is definitely incredible for paying for all of that. And, yeah, they're very awesome.” (Participant 21, 15-21 years old and completed treatment, Low toxicity (COST 28), Hodgkin lymphoma)  “But other than that, it really didn't [impact my parents’ financial situation] at all. Because of [institutional philanthropic funding].” (Participant 19, 15-21 years old and completed treatment, Moderate toxicity (COST 20), Acute lymphoblastic leukemia)  “So between [institutional philanthropic funding] helping take care of some of the medical bills… all my medications are paid for. And I have a l-- I'm on a lot of… medication. So anything I get from the [hospital]—[institutional philanthropic funding] covers it. So that was-- that's been really great. All the copays-- they pay for them.” (Participant 35, 15-21 years old and receiving treatment, Low toxicity (COST 37), Hodgkin lymphoma)  “I don't think it affected-- at [my hospital], they really provided my parents with as much as they could, in terms of gas because I had to drive an hour to get to the hospital from my home. And so they would give us gas vouchers. They would give us meal vouchers. They would give us even, like, I'm pretty sure they would just give us free stuff sometimes, just to make sure that we weren't struggling financially. There's been times where I've had meal vouchers. And I've had my dad bring up a whole bunch of stuff from the cafeteria down there.” (Participant 37, 15-21 years old and completed treatment, Low toxicity (COST 36), Ewing sarcoma)  “But we had [institutional philanthropic funding] there to cover most of it. So that was very helpful. We're grateful for that.” (Participant 39, 15-21 years old and completed treatment, Low toxicity (COST 34), Malignant mixed germ cell tumor (retroperitoneum))  “[Institutional philanthropic funding] has been absolutely wonderful during it. They've covered I think all of the cost of treatment if I remember correctly. So they've been amazing. And whatever they didn't cover… our insurance did.” (Participant 41, 15-21 years old and receiving treatment, Low toxicity (COST 43), Acute lymphoblastic leukemia)  “But thanks to [institutional philanthropic funding], we were to get along [financially] pretty well.” (Participant 3, 15-21 years old and receiving treatment, Low toxicity (COST 26), Acute lymphoblastic leukemia)  “… so I already, kind of, knew about [institutional philanthropic funding], because I did fundraisers for them from elementary through high school. But… it was more so my mom-- so my mom is a school nurse. And so when she-- she was in the hospital with me, I think a day after I had had surgery, she was talking with one of her friends, who is a special ed teacher at the school, and she told her, you know, like, she's already signed up for [institutional philanthropic funding]. You just need to discuss it with your doctors. And they all knew about [institutional philanthropic funding], and were very helpful with getting us set up, and already on the plan by the time, I think, I was discharged… it was really kind of a blessing. You know, mom and dad were stressing about all of the financial stress that that's going to put on, and how that would impact, you know, their ability to care for me. And just, the school called and said, [institutional philanthropic funding] will take care of it, and then insurance carried the rest of it.” (Participant 7, 15-21 years old and completed treatment, Low toxicity (COST 31), Juvenile pilocytic astrocytoma)  “… we had the [institutional philanthropic funding], which was amazing. That-- we were so thankful for that. It definitely took off any stressor that would have been there, you know what I mean? And then [my father’s] job provides good insurance as well. So that's nice as well. It's-- it didn't, like, have to enter our minds because of the [institutional philanthropic funding], which is amazing. Like, I think it definitely would have added a lot of stress if we didn't have that.” (Participant 9, 15-21 years old and completed treatment, Low toxicity (COST 30), Acute lymphoblastic leukemia)  “I was covered under the [institutional philanthropic funding]-- and so that was a big help, too… and then we could rely on that [institutional philanthropic funding]… for anything extra. So… that was a big, big help with all of that.” (Participant 11, 15-21 years old and completed treatment, Low toxicity (COST 30), Acute lymphoblastic leukemia)  “… honestly, I mean, [institutional philanthropic funding]'s being a backup and covering the rest of… my prescriptions and stuff… yeah, I have to go to physical therapy two or three times a-- a week… so I know they'll cover what my insurance doesn't cover. They've reimbursed us for a-- a shower head and a shower seat because I can't really stand in the shower, like, it takes everything out of me… so I know that they've been amazing and I really do think without them, this would be a harder battle, but I think everyone as of right now, can go to sleep knowing that my cancer stuff is taken care of by [institutional philanthropic funding].” (Participant 31, 15-21 years old and receiving treatment, Low toxicity (COST 33), Acute lymphoblastic leukemia)  “But I know our insurance did cover a lot. And obviously so did the [institutional philanthropic funding]. And so sometimes, I would actually look up the price of the different medications I was getting. And it was kind of crazy.” (Participant 37, 15-21 years old and completed treatment, Low toxicity (COST 36), Ewing sarcoma)  “They talked with my parents about the [institutional philanthropic funding] since they're the ones who handle, like, the financial stuff of everything. So I was in the room with them, but I don't understand what half of what they're saying is, like FMLA. I don't know what the heck that is, you know. Anything that is involved with finances at all just kind of goes over my head, and I say to myself. I don't have to worry about this. I'm not the one paying for it, you know.” (Participant 22, 15-21 years old and receiving treatment, Low toxicity (COST 41), Acute lymphoblastic leukemia)  “So they were-- like the, the first, my first month in the hospital, they sent up-- they sent us a bill to my house for an absurd amount of money. And we just took it down and my social worker's like, all right, that's fine. And we never saw a bill again. And, and there was just, I know for them that took a-- I mean, yes, they were stressed because their son had cancer. But [institutional philanthropic funding] took a lot of the stress off of them that they didn't have to worry about the finances, about it at all.” (Participant 19, 15-21 years old and completed treatment, Moderate toxicity (COST 20), Acute lymphoblastic leukemia) |
| **Subtheme 3b:** Participants cited alternative resources as a significant influence on their financial situation throughout their cancer journey. | “And then even [my mother’s] friends. I had a payment and they paid it off for me, stuff like that. Like anyone, all her friends pitched in to help her.” (Participant 13, 15-21 years old and completed treatment, Low toxicity (COST 37), Hodgkin lymphoma)  “I mean, we raised-- my mom raised a good bit of money. She did a fundraiser for me with t-shirts and everything else. And we-- pretty much everybody sold t-shirts, stickers, stuff like that. And that really helped. I think it ended up of the $8,000 or $9,000. So that, that really helped as far as treatments and all that.” (Participant 2, 22-25 years old and completed treatment, Low toxicity (COST 41), Hodgkin lymphoma)  “… that was [external funding] because I was-- like, I literally-- when I was diagnosed, I already had put money down to move into my own apartment. So, like, my mom, before the [external funding] started, like, helping me with rent, my mom had-- she moved with my sister so she could help me pay my bills because she knew I was looking forward to moving by myself. So, I guess, probably financially on my mom.” (Participant 8, 15-21 years old and completed treatment, Low toxicity (COST 27), Soft tissue sarcoma (angiosarcoma))  “… this sounds pretty ridiculous, but I got, like, a lot of money from relatives and stuff over the course of this. So I would probably use that to help fund [other expenses], obviously.” (Participant 22, 15-21 years old and receiving treatment, Low toxicity (COST 41), Acute lymphoblastic leukemia)  “And I-- you know, I had, you know, some government assistance, you know, to get… through, you know the food stamps all that other type of stuff. That helped too, of course.” (Participant 23, 22-25 years old and receiving treatment, Low toxicity (COST 29), Hodgkin lymphoma)  “But basically, I was receiving, like, money from the government. I was also receiving food stamps.” (Participant 34, 22-25 years old and completed treatment, Moderate toxicity (COST 22), Peripheral T cell lymphoma)  “I obviously do get pretty good scholarships for having cancer now, which is nice.” (Participant 37, 15-21 years old and completed treatment, Low toxicity (COST 36), Ewing sarcoma)  “And if I submitted W2, and so, like, when I wasn't working, like-- if you're under an income threshold essentially, you get a very big discount… on the, like, [fertility] preservation cost per year.” (Participant 1, 22-25 years old and completed treatment, Low toxicity (COST 29), Acute lymphoblastic leukemia) |
| **Subtheme 3c:** Participants cited both positive and negative experiences with health insurance throughout their cancer journey. | “I know my insurance definitely had made my mom played out of pocket a few times.” (Participant 13, 15-21 years old and completed treatment, Low toxicity (COST 37), Hodgkin lymphoma)  “… so my dad has kind of a crappy health insurance. I mean, like, the copays are pretty expensive, even with the health insurance.” (Participant 12, 15-21 years old and receiving treatment, Low toxicity (COST 26), Hodgkin lymphoma)  “So, like, I want to say, I'm really fortunate for, like-- because of [philanthropic institutional funding]. Plus, my dad-- I'm on, like, their insurance still. And my dad was military. So we have really great insurance, like, on top of [institutional philanthropic funding].” (Participant 35, 15-21 years old and receiving treatment, Low toxicity (COST 37), Hodgkin lymphoma)  “Thankfully, because my mom had insurance through her work and I was able to like remain on it because of my age, it ended up being OK. We didn't have any like serious debt because everything either got covered by her insurance and then-- not everything was covered, but then when I became eligible for Medicaid because I was like not working, then what we would have had in debt like got picked up by Medicaid. Because… Medicaid picks up like excessive debt, but if… for some reason that situation had not occurred in that way, like I had been on any insurance through my job or something, I would have been in a completely devastating financial situation. So I was like very lucky that it happened to work out.” (Participant 5, 22-25 years old and completed treatment, Low toxicity (COST 31), Acute lymphoblastic leukemia)  “… but we were under my dad's healthcare insurance, which is still really good at the time…. so that was a big help, you know, with him working on the railroad and having that good insurance. And then… the social worker that we had… she had also recommended that we try a secondary insurance to try to help cover, you know, some of those costs, and then we could rely on that [institutional philanthropic funding]… for anything extra.” (Participant 11, 15-21 years old and completed treatment, Low toxicity (COST 30), Acute lymphoblastic leukemia)  “And then insurance covered a lot. Insurance was what really amazed me. Because in the beginning, I think [my doctor] said that it was-- the one treatment was going to be like $300,000. He wanted to run it through insurance first. And then he said that my insurance approved it, [luckily]. So we thought still, I'd end up with a few thousand in bills. And I didn't. So that was very, very lucky on my end.” (Participant 2, 22-25 years old and completed treatment, Low toxicity (COST 41), Hodgkin lymphoma)  “For me… I was lucky. I had a job that provided me with insurance that covered the vast majority of my bills. But… I still took a look to see just how large those bills were that even insurance was getting. And the amounts were staggering… meaning that without my insurance, I would have had no help, clearing out all my funds, and there was-- severely hindering whatever my family may have.” (Participant 30, 22-25 years old and completed treatment, Low toxicity (COST 28), Testicular cancer)  “So I was immediately put onto Medicaid once I was diagnosed through a social worker at the hospital, which has been, I'm very grateful for that… because I think I would be-- possibly millions of dollars in debt without it… so that covered basically all of my treatment.” (Participant 36, 22-25 years old and completed treatment, Moderate toxicity (COST 25), Hodgkin lymphoma)  “But I know our insurance did cover a lot. And obviously so did the [institutional philanthropic funding]” (Participant 37, 15-21 years old and completed treatment, Low toxicity (COST 36), Ewing sarcoma)  “… it really… put a strain on [personal and family finances]… thankfully, I have pretty good insurance. So they were able to cover a lot of it.” (Participant 40, 15-21 years old and completed treatment, Moderate toxicity (COST 16), Hodgkin lymphoma)  “I just turned 26. I'm my own health care. I got this, like, I paid for the best, you know, plan that my-- my company offers…. So it's things like that where… they're-- you know, I-- I am fortunate that I didn't really have to cover any of the financial burden until I was off treatment…” (Participant 1, 22-25 years old and completed treatment, Low toxicity (COST 29), Acute lymphoblastic leukemia) |
| **Theme 4: Due to financial strain, some participants experienced stressful emotions (such as guilt, worry), while others did not.** | |
| **Subtheme 4a:** Some participants expressed guilt, stress, worry or other negative emotions about their financial situation due to cancer treatment. | “And, like, this just-- I felt bad because this was my burden, I guess.” (Participant 12, 15-21 years old and receiving treatment, Low toxicity (COST 26), Hodgkin lymphoma)  “So then we're just paying for everything again… so that was stressful.” (Participant 29, 22-25 years old and receiving treatment, Moderate toxicity (COST 24), Breast cancer)  “… during our move, there were-- there was definitely stressors… about finances. You know, nothing crazy… my dad has a very, you know, good job, thank God. So we weren't, you know, stressing over not paying the bills or anything. But when it came to, like, the move and, you know, the area that we moved to is a little bit more expensive than [the town] where we used to live. So that was just an adjustment, I think.” (Participant 9, 15-21 years old and completed treatment, Low toxicity (COST 30), Acute lymphoblastic leukemia)  “But there was no finan-- there was no financial stress towards anybody besides myself.” (Participant 23, 22-25 years old and receiving treatment, Low toxicity (COST 29), Hodgkin lymphoma)  “… I was constantly kind of stressed out knowing that I didn't have income and that I couldn't do anything about it, that I couldn't apply for anything” (Participant 40, 15-21 years old and completed treatment, Moderate toxicity (COST 16), Hodgkin lymphoma) |
| **Subtheme 4b:** Due to decreased financial stresses, some participants were able to focus on their professional goals, independence, and other pursuits. | “It was good to have the support system and not having to worry about rent or bills.” (Participant 13, 15-21 years old and completed treatment, Low toxicity (COST 37), Hodgkin lymphoma)  “Ah, I would guess that they just wanted to keep me as calm as, not have to worry about anything.” (Participant 15, 15-21 years old and completed treatment, Low toxicity (COST 36), Hodgkin lymphoma)  “[Institutional philanthropic funding] would take care of it, which was definitely really helpful for us, obviously, because we didn't have to worry about like the money. It was more about like what was going on, and like making sure I was OK.” (Participant 21, 15-21 years old and completed treatment, Low toxicity (COST 28), Hodgkin lymphoma)  “-- As much as they could, they wanted to kind of keep that away from me as to not have something on my plate to-- to worry about going through treatment. But, you know, having some line of sight into that, I-- I think at times it was challenging… And they did a very good job, like, keeping me out of it, uh, which it-- you know, I-- I, like, very much appreciated, but I also felt guilty about, you know? Like I was this like added expense where, at this point, like, I, you know, had hoped not to be… But Yes I would say their best efforts were in-- in keeping as much of that private as possible.” (Participant 1, 22-25 years old and completed treatment, Low toxicity (COST 29), Acute lymphoblastic leukemia)  “I'd say, right now, we're in a good spot… because we're doing everything through, like, [this institution].” (Participant 6, 15-21 years old and receiving treatment, Low toxicity (COST 35), Adrenocortical carcinoma)  “And what's nice about my situation is that… it's-- I don't have to get a job for college… everything is paid for. I don't need to worry about it. I'm-- just need to worry about education. That's all.” (Participant 41, 15-21 years old and receiving treatment, Low toxicity (COST 43), Acute lymphoblastic leukemia)  “It felt-- it-- it was-- it was good. Like, I was-- I felt more independent. I wasn't dependent on people. I could do what I wanted. I wasn't, like… stressing about money and stuff like that.” (Participant 8, 15-21 years old and completed treatment, Low toxicity (COST 27), Soft tissue sarcoma (angiosarcoma)) |
